# Impact of Glucagon-Like Peptide-1 Receptor Agonists on Knee Arthroplasty Outcomes

**DOI:** 10.1007/s43465-025-01646-5

**Published:** 2025-12-13

**Authors:** Haroon Zaffar, Mohammed Araiz Imran, Farwah Rushd

**Affiliations:** 1 East Lancashire NHS Trust, Blackburn, England, UK; 2 Royal Alexandra Hospital, Paisley, Scotland, UK; 3 University of Glasgow, Glasgow, Scotland, UK

**Keywords:** Total knee arthroplasty, Glucagon-like peptide-1 receptor agonists, Post-operative complications, Revision, 90-Day readmission

## Abstract

**Purpose:**

Glucagon-like peptide-1 receptor agonists (GLP-1 RAs) are increasingly used for managing both obesity and diabetes mellitus, two major risk factors for adverse outcomes following total knee arthroplasty (TKA). Despite this, their potential role in pre-operative optimisation remains uncertain. The aim of this systematic review and meta-analysis was to assess whether the use of GLP-1 RAs is associated with improved post-operative outcomes in patients undergoing primary TKA.

**Methods:**

A comprehensive literature search was conducted across MEDLINE, PubMed, Embase, and CENTRAL from inception to 1st June 2025. Eligible studies included adults (aged ≥ 18 years) undergoing primary TKA, comparing outcomes between GLP-1 RA users and non-users. Primary outcomes included surgical and medical complications. Secondary outcomes were hospital-related outcomes, including hospital readmissions and length of stay. Risk of bias and certainty of evidence were assessed using ROBINS-I and GRADE, respectively.

**Results:**

Six retrospective cohort studies met the inclusion criteria which consisted of 20,074 GLP-1 RA users and 55,332 controls. Meta-analysis showed a statistically significant reduction in the odds of 90-day readmissions in comparison with control groups (OR 0.76, 95% CI 0.61–0.94; *p* = 0.01). No statistically significant associations were found for the remaining outcomes.

**Conclusions:**

GLP-1 RA use was associated with reduced 90-day readmissions following TKA. However, significant heterogeneity limits the clinical applicability of these findings. Further prospective studies are warranted.

**Supplementary Information:**

The online version contains supplementary material available at 10.1007/s43465-025-01646-5.

## Introduction

Primary total knee arthroplasty (TKA) is amongst the most commonly performed orthopaedic procedures [1]. Projections based on the National Joint Registry estimated a 36.6% increase in TKA procedures in the United Kingdom, reaching 137,000 by 2060 [1]. A similar trend is seen internationally; in the United States (US), primary TKA volume is expected to increase by 469% over the same period, surpassing 1.2 million procedures yearly [2].

The primary indication for TKA is end-stage osteoarthritis (OA) [3], a degenerative joint condition strongly associated with modifiable risk factors, such as obesity and diabetes mellitus (DM) [4]. A modelling projection study in the UK predicted that the rates of obesity were projected to rise substantially by 2035 [5], this was paralleled by similar rising trends in DM [6, 7]. 

Obesity and DM are both recognised risk factors for adverse post-operative outcomes following TKA [8, 9]. Raju et al. demonstrated that patients with DM undergoing TKA were at higher risk of complications, such as periprosthetic joint infection (PJI), deep vein thrombosis (DVT), and hospital readmission [10]. Similarly, obesity has been associated with a greater risk of surgical site infections (SSI), revision surgery, and medical events [11]. Given these risks, optimising weight and glycaemic control in the pre-operative period is essential to reduce complications and improve outcomes [12, 13].

**Fig. 1:**
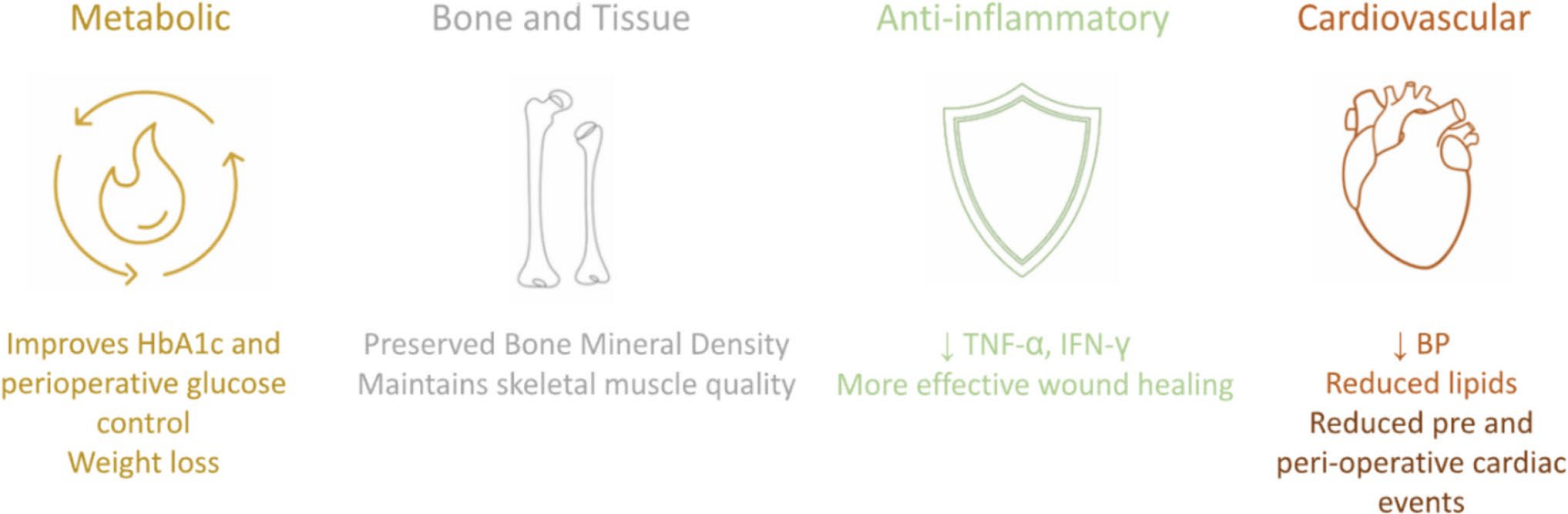
Proposed effects of GLP-RA on arthroplasty outcomes

Glucagon-like peptide-1 receptor agonists (GLP-1 RAs) have emerged as promising agents in this context [14, 15]. These agents were initially developed in the 1980s for DM management, based on research involving Gila monster venom [16]. Given their dual mechanism of action, they can additionally aid in weight loss. These agents work by mimicking endogenous incretin hormones to enhance glucose-dependent insulin secretion and suppress glucagon release to maintain glycaemic control. GLP-RAs also act on appetite-regulating pathways and delay gastric emptying to contribute to weight loss [17]. Randomised control trials have shown that GLP-1 RAs can induce significant weight loss while also improving glycaemic control [18, 19]. Several mechanisms by which GLP-1 RAs may influence arthroplasty outcomes are illustrated in Fig. 1.

Several studies have investigated the effects of other weight loss methods and alternative diabetic medications on outcomes following TKA [12, 20, 21]. However, to the best of our knowledge, no reviews exist investigating the impact of GLP-1 RA use on surgical and medical complications as well as hospital-related outcomes following primary TKA. This review was conducted to address this gap in the evidence. 

## Materials and Methods

This review was conducted in accordance with the Methodological Expectations of Cochrane Intervention Reviews (MECIR) standards and was reported in adherence with the Preferred Reporting Items for Systematic Reviews and Meta-Analyses (PRISMA) 2020 guidelines [22, 23]. This review was registered within the PROSPERO database (CRD420251084068).

### Eligibility Criteria

Criteria followed the PICO (population, intervention, comparator, and outcome) framework. Included studies were in English and assessed the use of GLP-1 RAs and their impact on TKA. Observational studies and both randomised and non-randomised trials were eligible. Given the differences in length of exposure in each study; studies were eligible if the timing and duration of GLP-1 RA therapy was likely to impact post-operative outcomes. Case reports, abstracts without full-text, editorials and non-peer review publications were excluded.

The population included adults (aged ≥ 18 years) undergoing primary TKA; revision cases were excluded. The indication of GLP-1 RA use (DM or obesity) was not restricted. Comparators were patients undergoing TKA without GLP-1 RA exposure. Primary outcomes consisted of both medical (deep vein thrombosis (DVT), pulmonary embolism (PE), acute kidney injury (AKI) and pneumonia) and surgical complications (periprosthetic joint infection (PJI), periprosthetic fracture (PPF) and revision surgery). Secondary outcomes investigated hospital-related outcomes, including readmissions and length of stay (LOS).

### Search Strategy and Information Sources

We carried out a systematic literature search from the date of inception to 1st June 2025 using PubMed, Embase, and the Cochrane Central Register of Controlled Trials (CENTRAL) databases to identify eligible studies. Our search strategy to identify these papers included the use of terms relating to GLP-1 RA, TKA and its relevant outcomes. Full search strategy has been provided with Supplementary File Table S1.

### Selection Process

Three reviewers independently carried out full-text reviews of the relevant studies matching the eligibility criteria. Any duplicate findings or disagreements arising during the selection process were resolved with discussion.

### Data Collection Process

Two reviewers independently extracted data into a Microsoft Excel spreadsheet, including study design, patient demographics, GLP-1 RA agent, timing relative to surgery and all reported outcomes. Outcomes were categorised by as short-term (≤ 90 days) or long-term (≥ 1 year). When only percentages were reported, absolute event counts were calculated using the total sample size. Discrepancies were resolved through discussion. Where data were missing or unclear, study authors were contacted for clarification.

### Risk-of-Bias Assessment

Risk-of-bias assessment was conducted by two independent reviewers using Cochrane Risk-of-bias 2 (RoB-2) tool for randomised trials and the risk of bias in non-randomized studies of interventions (ROBINS-I) tool for observational studies [24, 25]. Each study was graded as either low, moderate, serious or critical. Any discrepancies were resolved with discussion.

### Reporting Bias Assessment

Where available, study protocols were compared to reported outcomes. Publication bias was assessed using funnel plots and Egger’s test for outcomes with data from ten or more studies. For outcomes with fewer than ten studies—a formal publication bias assessment was not conducted.

### Certainty Assessment

Two reviewers rated the certainty of evidence using the GRADE (Grading of Recommendation, Assessment, Development and Evaluation) tool. This tool is based off the following five domains: risk of bias, inconsistency, indirectness, imprecision and potential publication bias [26]. All ratings and their justifications were recorded in a Summary of Findings table (Table 2).

### Data Synthesis and Statistical Analysis

ReviewManager software (Version 5.4.1) software was used to conduct a meta-analysis. Dichotomous outcomes were synthesised as odds ratio (OR) all with 95% confidence intervals (CIs) using a random-effects model. Between-study variance was estimated using the DerSimonian and Laird method. Statistical heterogeneity was quantified using both the Chi^2^ and the *I*^2^ tests. According to the Cochrane Handbook, *I*^2^ values of 0–40% may indicate minimal heterogeneity, 30–60% suggest moderate heterogeneity, 50–90% indicates substantial heterogeneity, and 75–100% reflect considerable heterogeneity [22]. A *p* value of < 0.05 was considered statistically significant. Sensitivity analyses were conducted to account for unclear event counts. Planned subgroup analyses were based on GLP-1 RA type and diabetic status, if data permitted. Where data were not comparable across studies, findings were described narratively.

## Results

### Search Result

**Fig. 2:**
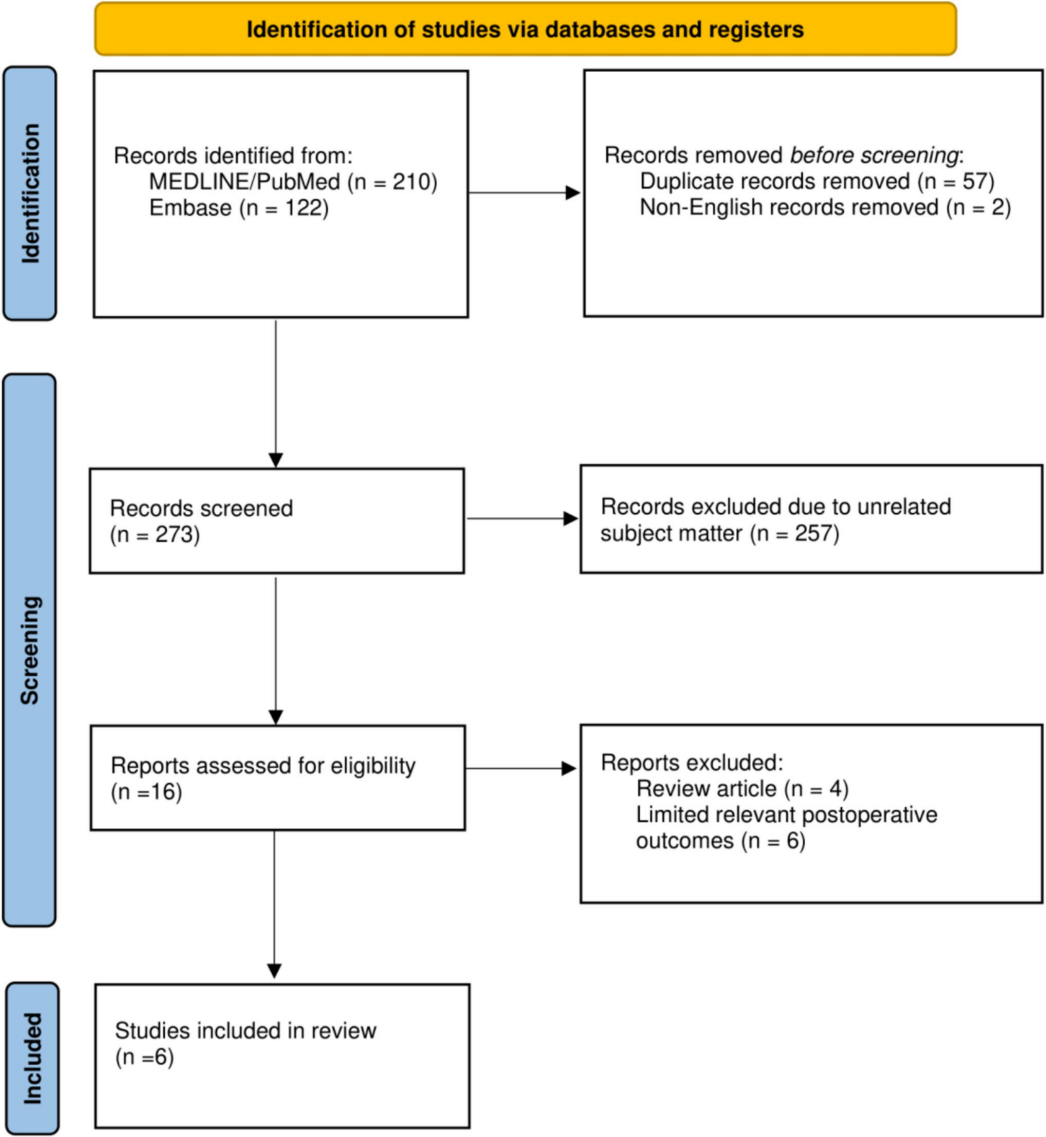
Preferred Reporting Items for Systematic Reviews and Meta-Analyses (PRISMA) flow diagram of study search and inclusion

The search strategy identified a total of 332 records across all databases. 57 duplicates and two non-English records were removed. Title and abstract screening led to the exclusion of 257 records that clearly did not meet the inclusion criteria. 16 full-text articles were retrieved and assessed for eligibility. Of these, four were review articles, and six had limited reporting of relevant post-operative outcomes. Six studies met the inclusion criteria and were included in the qualitative and quantitative synthesis [27–32]. The study selection process is summarised in the PRISMA 2020 flow diagram (Fig. 2).

### Characteristics of Included Studies

All six included studies were matched retrospective cohort analyses conducted in the US, with sample sizes ranging from 4190 to 41,575 participants [27–32]. In total, 20,074 patients received GLP-1 RA and 55,332 served as matched controls. Where reported, the mean age ranged from 61 to 64.2 years, and the proportion of male participants was similar across groups, ranging from approximately 33.1–43.6%. All studies assessed various outcomes, including medical and surgical complications, at different follow-up intervals—typically at 90 days, 1 year, or 2 years post-operatively. Table 1 summarises these characteristics.

**Table 1:** Study characteristics

Author and year	GLP-1 (*n*)	Control (*n*)	Mean age (year)		Male *N* (%)		GLP-1 RA agent	Outcomes assessed
			GLP-1	Control	GLP-1	Control		
Buddhiraju 2024 [27]	2095	2095	64.1	64.2	693 (33.1)	683 (32.6)	NR	90-day medical, 90-day revision
Heo 2024 [28]	2388	2388	61.2	61	1013 (42.4)	1040 (43.6)	NR	90-day medical and surgical, 1-year surgical
Katzman 2025 [29]	865	8650	64	64	294 (34)	2911 (33.7)	Semaglutide, Liraglutide, Dulaglutide, Exenatide, Tirzepatide, Lixisenatide, Albiglutide	2-year revision
Kim 2025 [30]	2975	2975	62.2	62.2	989 (33.2)	986 (33.1)	Exenatide (including microsphere formulation), Semaglutide, Dulaglutide, Liraglutide	90-day medical and surgical, 2-year surgical
Levidy 2025 [31]	4700	4700	NR		NR		Liraglutide, Pramlintide, Tirzepatide, Semaglutide, Lixisenatide, Sulaglutide	90 days and 1-year surgical outcomes
Magruder 2023 [32]	7051	34,524	^a^	^a^	2720 (38.6)	13,265 (38.4)	Semaglutide	90-day medical, 2-year surgical

^a^In Magruder et al., the number (%) of patients in each age category in the GLP-1 RA vs control cohorts were as follows: 40–44 years, 25 (0.4%) vs 106 (0.3%); 45–49 years, 215 (3.0%) vs 1,012 (2.9%); 50–54 years, 739 (10.5%) vs 3,563 (10.3%); 55–59 years, 1,376 (19.5%) vs 6,770 (19.6%); 60–64 years, 1,879 (26.6%) vs 9,268 (26.8%); 65–69 years, 1,502 (21.3%) vs 7,383 (21.4%); 70–74 years, 955 (13.5%) vs 4,699 (13.6%); and 75–79 years, 339 (4.8%) vs 1,631 (4.7%). No patients were reported in age categories above 79 years

### Medical Complications

#### Deep Vein Thrombosis

**Fig. 3:**
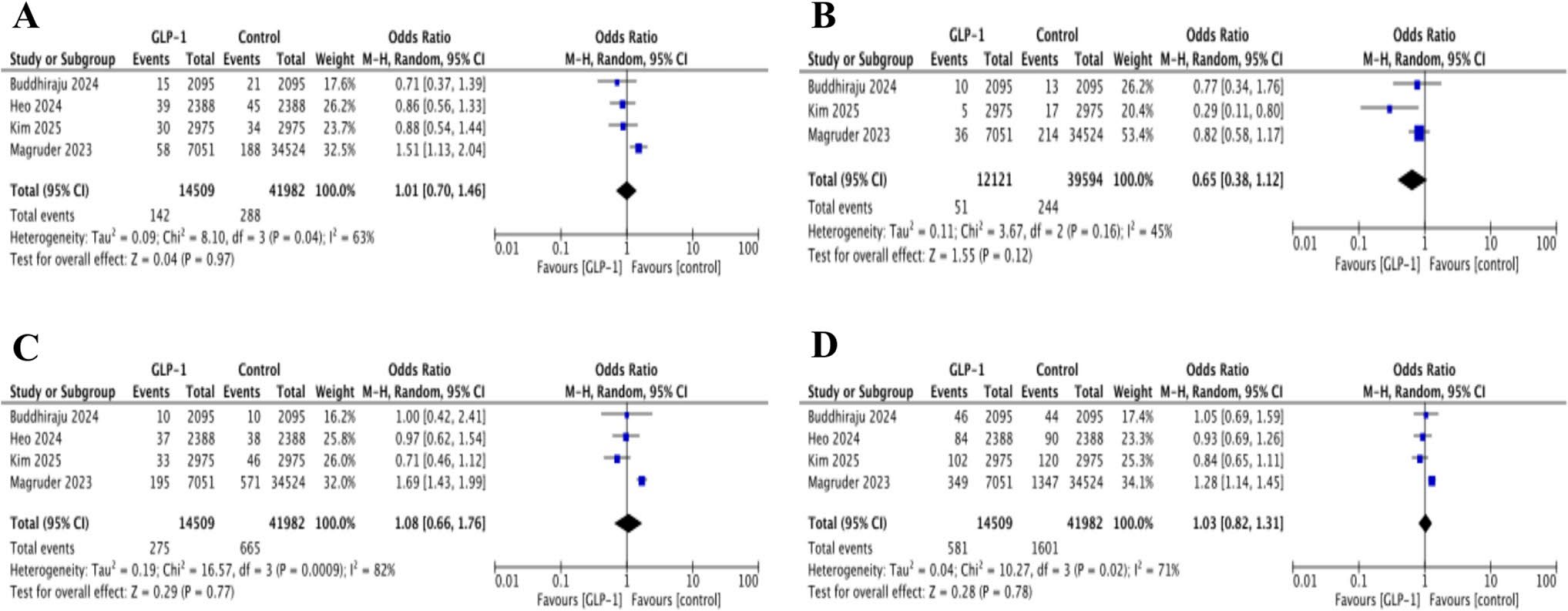
Forest plots showing pooled effect estimates for: **A** deep vein thrombosis, **B** pulmonary embolism, **C** pneumonia, **D** acute kidney injury

Four studies reported 90-day post-operative DVT events [27, 28, 30, 32]. The pooled analysis demonstrated no statistically significant difference between the GLP-1 RA and control groups [OR 1.01, 95% CI 0.70–1.46, *p* = 0.97] with substantial heterogeneity [*I*^2^ = 63%] (Fig. 3).

#### Pulmonary Embolism

Three studies evaluated 90-day PE events [27, 30, 32]. The combined results displayed no significant difference between groups [OR 0.65, 95% CI 0.38–1.12; *p* = 0.12; *I*^2^ = 45%] (Fig. 3).

#### Pneumonia

Four studies assessed the occurrence of 90-day pneumonia [27, 28, 30, 32]. Analysis found no significant difference (OR 1.08, 95% CI 0.66–1.76; *p* = 0.77), although heterogeneity was considerable (*I*^2^ = 82%) (Fig. 3).

#### Acute Kidney Injury

AKI events within 90 days were reported in four studies [27, 28, 30, 32]. Findings were inconclusive, with no statistically significant difference observed (OR 1.03, 95% CI 0.82–1.31; *p* = 0.78; *I*^2^ = 71%) (Fig. 3).

#### Sensitivity Analysis for Medical Complications

High levels of heterogeneity were observed for several medical outcomes, including DVT, pneumonia and AKI. Sensitivity analyses were performed by excluding the study by Magruder et al., which consistently contributed the largest weight across these analyses [32]. Heterogeneity in all three outcomes reduced to 0%. The exclusions also shifted the pooled estimates towards a non-statistically significant reduction in risk, favouring GLP-1 RA users. These findings suggest that the original pooled results were heavily influenced by the inclusion of Magruder et al.

### Surgical Complications

#### Periprosthetic Joint Infection

**Fig. 4:**
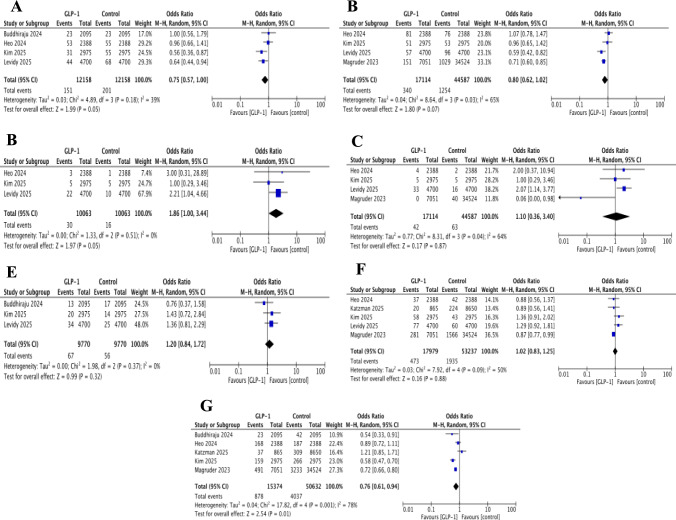
Forest plots showing pooled effect estimates for: **A** short-term periprosthetic joint infection, **B** long-term periprosthetic joint infection, **C** short-term periprosthetic fracture, **D** long-term periprosthetic fracture, **E** short-term revision surgery, **F** long-term revision surgery, and **G** 90-day hospital readmission

Four studies were included in the analysis of short-term PJI [27, 28, 30, 31]. The pooled analysis showed no statistically significant difference between groups (OR 0.75, 95% CI 0.57–1.00; *p* = 0.05; *I*^2^ = 39%) (Fig. 4).

Long-term PJI outcomes were reported in four studies [28, 30–32]. While the pooled analysis did not reach statistical significance, there was a slight trend towards reduced risk with GLP-1 RA use (OR 0.80, 95% CI 0.62–1.02; *p* = 0.07; *I*^2^ = 65%) (Fig. 4).

In both analyses, the study by Heo et al. contributed considerable weight and reported a near-null effect [28], differing from the protective associations observed in the other studies [27, 29–32]. Sensitivity analyses excluding Heo et al. resulted in statistically significant results [28]. Specifically short-term PJI yielded a pooled OR of 0.68 (95% CI 0.50–0.91; *p* = 0.009; *I*^2^ = 20%) and in a similar manner long-term PJI was associated with OR of 0.72 (95% CI 0.58–0.90; *p* = 0.004; *I*^2^ = 44%). The near-null effect reported by Heo et al. may reflect strict exposure definitions requiring high prescription adherence, potentially misclassifying some users and diluting the effect. In addition, rigorous matching and adjustment for DM severity and comorbidities may have minimised group differences, making true effects harder to detect. A possible protective effect is suggested, but confirmation through prospective studies is warranted.

#### Periprosthetic Joint Fracture

Three studies contributed data on short term-PPF [28, 30, 31]. The pooled analysis did not show a statistically significant difference between GLP-1 RA users and controls (OR 1.86 95% CI 1.00–3.44; *p* = 0.05), with no observed heterogeneity (*I*^*2*^ = 0%) (Fig. 4).

However, the analysis was highly sensitive to several factors. The overall result was largely driven by Levidy et al., which contributed most of the study weight [31]. Furthermore, Heo et al. reported highly imprecise estimates with wide confidence intervals, likely reflecting sparse data [28]. Collectively, these issues indicate that the findings for this outcome are unstable.

Long-term PPF was also reported within four studies with follow-up ranging from 1 to 2 years [28, 30–32]. The pooled analysis found no statistical difference between the two groups (OR 1.10 95% CI 0.36–3.40; *p* = 0.79 *I*^*2*^ = 64%) (Fig. 4).

#### Revision

Short-term revision was assessed in four studies with no significant difference observed between groups (OR 1.20, 95% CI 0.84–1.72; *p* = 0.32; *I*^2^ = 0%) [28, 30–32]. Similarly, five studies reporting long term revision over 1–2 years of follow-up also found no statistically significant difference (OR 1.02, 95% CI 0.83–1.25; *p* = 0.88; *I*^2^ = 50%) [28–32] (Fig. 4**).**

### Secondary Outcomes

#### 90-Day Readmissions

Five studies reported on 90-day hospital readmission rates [27–30, 32]. GLP-1 RA use was associated with a statistically significant reduction in the odds of readmission compared to controls (OR 0.76, 95% CI 0.61–0.94; *p* = 0.01). Although heterogeneity was considerable (*I*^2^ = 78%). Sensitivity analyses excluding the study by Magruder et al. resulted in a loss of statistical significance and an increase in heterogeneity (*I*^2^ = 83%) [32] (Fig. 4). A potential explanation is that Magruder et al. included only DM patients—a higher risk group in whom GLP-1 RA may more effectively reduce readmissions than in border population. Its large analytic weight may have driven the pooled effect and masked variability among smaller studies, indicating the observed readmission benefit should be interpreted cautiously.

#### Length of Stay

LOS was reported with considerable heterogeneity across studies and hence was not suitable for meta-analysis. Overall, LOS was reported to be shorter in the GLP-1 RA group, although the degree of difference varied. Magruder et al. reported a shorter average LOS in the GLP-1 RA group (2.7 days) compared to the control (3.1 days); however, no standard deviations (SD) were provided [32]. Kim et al. reported a mean LOS of 2.9 days (SD 1.8) in the control when compared to 2.7 days (SD 2.3) LOS in the GLP-1 RA group [30]. Heo et al. reported that 607 (25.4%) patients in the GLP-1 RA group experienced stays longer than 3 days, compared to 746 (31.2%) patients in the comparison group (OR 1.29, 95% CI 1.14–1.47; *p* < 0.001) [28]. Similarly, Katzman et al. reported a greater mean LOS in the control group (2.5 days; range 0.3–19.5) compared with the GLP-1 RA cohort (2.1 days; range 0.3–13.4) [29].

#### Risk-of-Bias Assessment

**Fig. 5:**
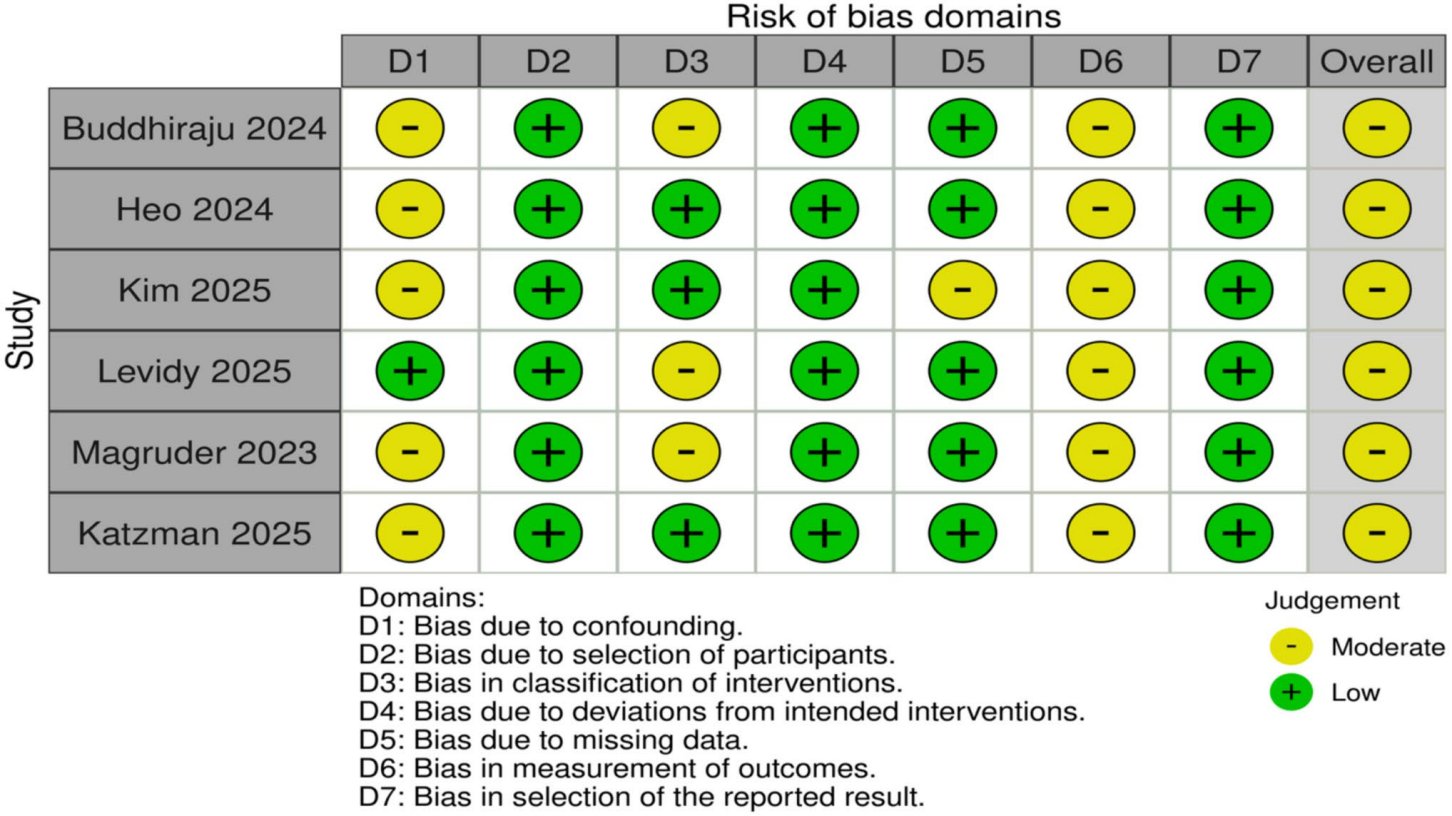
Risk-of-bias summary using the ROBINS-I tool for all included studies

All six studies were judged to have a moderate overall risk of bias [27–32]. A summary of the risk-of-bias assessments is provided in Fig. 5, with detailed individual justifications presented in the Supplementary File Table S2.

#### Certainty of Evidence

Four outcomes were assessed as having very low certainty using GRADE [26]. These outcomes were selected to reflect a balanced representation of medical and surgical complications as well as hospital-related measures. Downgrades were primarily due to risk of bias inherent in the retrospective study designs, with further downgrading for imprecision and inconsistency. The full assessments are presented in the Summary of findings table (Table 2).

**Table 2:**
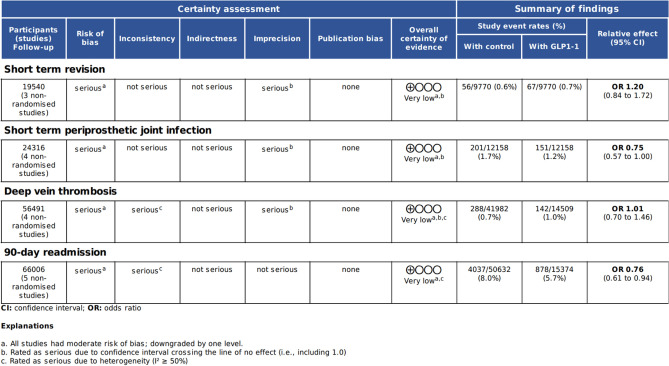
Summary of findings from GRADE assessment

#### Reporting Bias

Formal assessment of reporting bias (e.g., funnel plots) was not performed due to fewer than 10 studies per outcome, consistent with Cochrane guidance [22]. Where study protocols were available for comparison, no evidence of selective outcome reporting was identified.

## Discussion

This systematic review and meta-analysis found that GLP-1 RA use was associated with a statistically significant reduction in 90-day readmissions following TKA, although the certainty of evidence was very low. No statistically significant associations were identified for other medical or surgical complications. While a meta-analysis of LOS was not feasible, all studies reporting LOS reported a shorter LOS among GLP-1 RA users. Several plausible mechanisms may explain the observed results.

GLP-1 RAs improves glycaemic control through several mechanisms, including enhancing insulin secretion, reducing glucagon levels and delaying gastric emptying [17]. Elevated HbA1c levels are associated with higher risks of surgical site infections (SSI) and delayed wound healing [33]. Given that SSI is the major cause of remission following primary TKA, as identified by Ramkumar et al., it is plausible that improved glycaemic control may have contributed to lower infection-related admission in this cohort [34]. In addition, emerging evidence suggests that GLP-1 RAs may exert immunomodulatory effects by suppressing key inflammatory cytokines, such as TNF-α and IFN-γ, potentially lowering infection risk by supporting more effective wound healing [35].

However, this potential benefit was not clear in the SSI outcomes reported by the included studies. Meta analysis was not feasible due to limited reporting. Heo et al. reported similar 90-day SSI rates between GLP-1 RA users (4.7%) and controls (4.5%) (OR 0.95, 95% CI 0.73–1.26; *p* = 0.74) [28], while Buddhiraju et al. also found no significant difference (0.6% vs 0.5%; *p* = 0.525) [27]. The impact of GLP-1 RAs on SSI rates following TKA remains uncertain based on current evidence.

A recent review by Chan et al. accessed the effects of GLP-1 RA agents on surgical complications following joint arthroplasty [36]. While our findings were broadly consistent regarding short and long-term risks of PPF and revision, a key difference emerged in the analysis of PJI risk. Unlike Chan et al. which reported a statistically significant reduction in short and long-term PJI risk, our analysis found no statistically significant association. This discrepancy likely reflects important methodological differences. Chan et al. combined hip and TKA data without performing a knee-specific subgroup analysis. In contrast, our review focused exclusively on TKA, providing a more focussed evaluation of knee-specific outcomes. Moreover, we included two recent studies—Levidy et al. and Katzman et al.—with the former providing additional short- and long-term PJI data [29, 31].

### Limitation of the Evidence

There were several limitations within the included evidence. All studies relied on administrative or electronic health records, which are prone to outcome misclassification and potential under-reporting—factors that may have contributed to the lack of significant findings for some outcomes.

Exposure definitions varied considerably. Katzman et al. defined GLP-1 RA use as active use within 6 months before surgery and continued for 3 months after [29]. In contrast, Magruder et al. classified exposure on having a prescription at the time of surgery, without clear information on duration [32]. Such variability in exposure definitions, along with limited reporting on duration and dosing, complicates the interpretation of the observed associations. In addition, none of the included studies reported whether GLP-1 RAs were withheld prior to surgery. This is relevant given the guidance from the American Society of Anesthesiologists, which recommends withholding daily GLP-1 RAs on the day of surgery and weekly agents for 1 week prior, due to concerns about delayed gastric emptying and aspiration risk [37]. Although all included studies were conducted in the US, peri-operative protocols may have varied between centres, potentially introducing heterogeneity in glycaemic control and related outcomes.

There was also considerable heterogeneity in study populations. Three studies included only DM patients [28, 31, 32], whereas others comprised cohorts with varying proportions of DM and obesity [27, 29, 30]. This raises concerns about confounding by indication, as GLP-1 RA dosing varies by treatment intent [38]. Limited demographic diversity may also restrict generalisability of findings. Finally, agent-specific data were also limited: only Magruder et al. focused solely on semaglutide [32], while Katzman et al. reported agent use without outcome stratification [29]. Collectively, these limitations constrained the ability to conduct meaningful subgroup analyses.

Further limitations were noted in Kim et al., which used the TriNetX database, where event counts below 11 are suppressed to maintain patient confidentiality [30]. Several outcomes were reported as “ < 11,” introducing potential uncertainty into pooled estimates. In the absence of author clarification, a midpoint value of five was assumed. Sensitivity analyses using conservative (*n* = 1) and liberal (*n* = 10) estimates demonstrated minimal impact on the overall results.

This review was limited by the available evidence. Only retrospective studies were included, restricting the ability to infer causality and introducing potential confounding, information and selection bias. The focus on English-language publications from selected databases may have excluded relevant research. Variations in reporting, particularly for LOS, prevented meta-analyses, and several outcomes could not be assessed due to limited reporting among studies. As such, certain relevant findings may not be captured in this review**.**

## Conclusion

In summary, GLP-1 RA use was associated with a statistically significant reduction in 90-day readmission following TKA, while no differences were observed for other medical or surgical complications. Given the limitations of this review, high-quality prospective studies are needed to confirm these findings.

## Supplementary Information

Below is the link to the electronic supplementary material.

Supplementary file1 (PDF 353 KB)

## Data Availability

All data used in this study were extracted from previously published studies that are publicly available. A full list of sources is provided in the References section.
