# Supplementary material for: Impact of Glucagon-Like Peptide-1 Receptor Agonists on Knee Arthroplasty Outcomes
Source: Indian J Orthop. 2025 Dec 13;60(3):612–22. doi: 10.1007/s43465-025-01646-5 (PMC13031487; doi:10.1007/s43465-025-01646-5)
Supplement: Supplementary file 1 — Supplementary file1 (PDF 353 KB) [file 43465_2025_1646_MOESM1_ESM.pdf]

Supplementary Information

Article Title - Impact of glucagon-like peptide-1 receptor agonists on knee arthroplasty outcomes

Journal name - Indian Journal of Orthopaedics

Supplementary Table S1. Full electronic search strategy for each database as of [01/06/2025]

Database	Search Strategy
**Medline (via PubMed)**	("Arthroplasty, Replacement, knee"[Mesh] OR "knee Prosthesis"[Mesh] OR "knee Arthroplasty, Total"[Mesh] OR "knee arthroplasty"[tiab] OR "knee replacement"[tiab] OR "total knee arthroplasty"[tiab] OR "TKA"[tiab])<br>AND<br>("Glucagon-Like Peptide 1"[Mesh] OR "Glucagon-Like Peptide 1 Receptor"[Mesh] OR "GLP-1 receptor agonist"[tiab] OR "GLP-1"[tiab] OR "incretin mimetic"[tiab] OR "semaglutide"[tiab] OR "liraglutide"[tiab] OR "dulaglutide"[tiab] OR "exenatide"[tiab] OR "lixisenatide"[tiab])<br>AND<br>("Postoperative Complications"[Mesh] OR "Surgical Wound Infection"[Mesh] OR "readmission"[tiab] OR "revision"[tiab] OR "reoperation"[tiab] OR "infection"[tiab] OR "infections"[tiab] OR "periprosthetic fracture"[tiab] OR "periprosthetic joint infection"[tiab] OR "PJI"[tiab]OR "medical complications"[tiab])
**Embase**	('knee arthroplasty'/exp OR 'knee prosthesis'/exp OR 'total knee arthroplasty':ti,ab OR 'knee arthroplasty':ti,ab OR 'knee replacement':ti,ab OR TKA:ti,ab)<br>AND<br>('glucagon like peptide 1'/exp OR 'glucagon like peptide 1 receptor agonist'/exp OR 'glucagon like peptide 1 receptor':ti,ab OR 'glp-1 receptor agonist':ti,ab OR 'GLP-1':ti,ab OR 'incretin mimetic':ti,ab OR semaglutide:ti,ab OR liraglutide:ti,ab OR dulaglutide:ti,ab OR exenatide:ti,ab OR lixisenatide:ti,ab)<br>AND<br>('postoperative complication'/exp OR 'surgical wound infection'/exp OR readmission:ti,ab OR revision:ti,ab OR reoperation:ti,ab OR infection:ti,ab OR infections:ti,ab OR 'periprosthetic fracture':ti,ab OR 'periprosthetic joint infection':ti,ab OR PJI:ti,ab OR 'medical complication':ti,ab)
**Cochrane Central Register of Controlled Trials (CENTRAL)**	(knee arthroplasty OR knee replacement OR total knee arthroplasty OR knee prosthesis OR TKA)<br>AND<br>(GLP-1 OR GLP-1 receptor agonist OR glucagon like peptide 1 OR glucagon like peptide 1 receptor OR incretin mimetic OR semaglutide OR liraglutide OR dulaglutide OR exenatide OR lixisenatide)<br>AND<br>(postoperative complication OR surgical wound infection OR infection OR infections OR readmission OR revision OR reoperation OR periprosthetic fracture OR periprosthetic joint infection OR PJI OR medical complication)

Supplementary Table S2. ROBINS-I risk of bias assessments with justifications for each included study

Study	ROBINS-I Domain							
	**Confounding**	**Selection of participants**	**Classification of interventions**	**Deviations from intended interventions**	**Missing data**	**Measurement of outcomes**	**Selection of reported result**	**Overall**
**Buddhiraju 2024**	Did not match for BMI as continuous variable	Participants were selected based on pre intervention characteristics, with selection unlikely to be influenced by outcome status	Included if prescription recorded from 1 year to 15 days pre-op; may reflect short past use rather than sustained exposure	Retrospective design with no evidence of differential deviations from the intended intervention between groups	No indication of missing outcome data; relevant endpoints appeared complete and consistently reported across groups	Outcomes were derived from administrative or electronic health records, with potential for misclassification	All prespecified outcomes and time points were reported; where available, protocols were reviewed. No evidence of selective reporting	Moderate
**Heo 2024**	Key confounders such as diabetes severity (hba1c) and BMI as continuous variable were not counted for		Exposure defined as ≥3 fills in 6 months or ≥1 90-day fill pre-op ensured reliable perioperative exposure					Moderate
**Kim 2025**			Defined as minimum three months pre- and post-op use, ensuring clear perioperative exposure		Although data was not missing multiple outcomes were reported as <11, obscuring the exact values			Moderate
**Levidy 2025**	Propensity score matching included key confounders such as BMI (categorised) and HbA1c, reducing risk of residual confounding		Included if prescription recorded 12 month prior to surgery; may reflect short past use rather than sustained exposure		No indication of missing outcome data; relevant endpoints appeared complete and consistently reported across groups			Moderate
**Magruder 2023**	Key confounders such as diabetes severity (hba1c) and BMI as continuous variable were not counted for		Exposure defined as having GLP - 1RA prescription at surgery; duration unclear, raising some misclassification risk					Moderate
**Katzman 2025**			Perioperative GLP-1RA use is clearly defined as active use within 6 months before and continued within 3 months after surgery.					Moderate

Low **Moderate** **Serious**
